# Bark anatomy, chemical composition and ethanol-water extract composition of *Anadenanthera peregrina* and *Anadenanthera colubrina*

**DOI:** 10.1371/journal.pone.0189263

**Published:** 2017-12-27

**Authors:** Graciene S. Mota, Caroline J. Sartori, Isabel Miranda, Teresa Quilhó, Fábio Akira Mori, Helena Pereira

**Affiliations:** 1 Universidade Federal de Lavras, Departamento de Ciências Biológicas, Lavras, MG, Brazil; 2 Universidade Federal de Lavras, Departamento de Ciências Florestais, Lavras, MG, Brazil; 3 Centro de Estudos Florestais, Instituto Superior de Agronomia, Universidade de Lisboa, Lisboa, Portugal; University of Vigo, SPAIN

## Abstract

The bark of *Anadenanthera peregrina* (L.) Speg and *Anadenanthera colubrina* (Vell.) Brenan were characterized in relation to anatomical and chemical features. The barks were similar and included a thin conducting phloem, a largely dilated and sclerified non-conducting phloem, and a rhyridome with periderms with thin phellem interspersed by cortical tissues. Only small differences between species were observed that cannot be used alone for taxonomic purposes. The summative chemical composition of *A*. *peregrina* and *A*. *colubrina* was respectively: 8.2% and 7.7% ash; 28.8% and 29.3% extractives; 2.4% and 2.6% suberin; and 18.9% lignin. The monosaccharide composition showed the predominance of glucose (on average 82% of total neutral sugars) and of xylose (9%). The ethanol-water extracts of *A*. *peregrina* and A. *colubrina* barks included a high content of phenolics, respectively: total phenolics 583 and 682 mg GAE/g extract; 148 and 445 mg CE/g extract; tannins 587 and 98 mg CE/g extract. The antioxidant activity was 238 and 269 mg Trolox/g extract. The barks of the *Anadenanthera* species are a potential source of polar extractives that will represent an important valorization and therefore contribute to improve the overall economic potential and sustainability of *A*. *peregrina* and *A*. *colubrina*

## Introduction

Anadenanthera is a small neotropical genus from South America with only two species: *Anadenanthera peregrina* (L.) Speg and *Anadenanthera colubrina* (Vell.) Brenan [1]. They belong to the family Leguminosae, subfamily Mimosaceae, and can grow in areas from savannah to dry rainforest. In Brazil, where they are usually named “angico”, they occur predominantly in seasonal forests and riparian galleries [2, 3, 4].

Both species have a strong morphological similarity and their differentiation usually requires the examination of flowers with buds, fruits and leaves [1, 4]. The bark may provide taxonomic information when these bodies are not available either in herbaria or in the field. Although the external macroscopic characteristics of barks is important and is often used for identification, in the case of similar species, as it is the case of *A*. *peregrina* and *A*. *colubrina*, it is necessary to have a microscopic knowledge of the internal structure [5]. This has been shown by e.g. [6] for several *Eucalyptus* species, [7] who distinguished six bark types within the Cassinoideae subfamily, and [8] to distinguish Lebeckia genera.

Both *A*. *peregrina* and *A*. *colubrina* are used for timber, charcoal and as firewood, as well as in popular medicine [9]. The wood has high density, resistance and outdoor durability, as well as a smooth lustrous surface with high decorative value, and is used for flooring and in building and naval construction [10, 3].

Very few studies have been published on the stem characterization of the species of this genus. The wood anatomy and some physical properties were described for *A*. *peregrina* [10, 11] and the wood anatomy for *A*. *colubrina* [12]. As regards bark anatomy, [13] gave information of anatomy and hystochemistry of secretory ducts in the bark of A. peregrina, [14] and [15] gave general information of various genus of Leguminosae, and [16] examined 28 species of the subfamily Caesalpinioideae, Mimosaceae and Papilionoideae from Brazil, including the genus *Adenanthera* and *A*. *peregrina*.

More information exists on some chemical features of the barks of these species, since they have a high content of tannins that are used for the leather and dyeing industries (17, 18, 19, 4]. They are also used in the popular medicine for treatment of respiratory and lung infections, and against diarrhea [20]. Some phytochemical, pharmacological and toxicological studies of bark extracts and tannin quantification have been made for *A*. *peregrina* [21, 22] and *A*. *colubrina* [23, 24, 25, 26, 27].

Barks are a subject of recent research on their structural and chemical characterization targeting their potential as a feedstock for biorefineries. Barks from various species were studied e.g. *Eucalyptus* spp. [28, 29, 30], *Pseudotsuga menziesii* [31], *Quercus cerris* [32], *Tectona grandis* [33]. Overall it has been shown that knowledge on the anatomy and chemical composition of the barks is essential for designing their fractionation and valorization routes.

The barks of *A*. *peregrina* and *A*. *colubrina* are studied here for the first time with a comprehensive characterization of their structural and anatomical features as well as of the chemical composition, including polar extracts and their antioxidant properties.

The objective is dual: i) to see if the detailed information on the bark may be used as a taxonomical tool for species identification; and ii) to establish a background information for bark valorization to be used for targeting processing routes and products.

## Material and methods

No specific permits were required for the collection of bark of the species studied, since these were collected in the experimental field of the Federal University of Lavras and the non-destructive method was used. The field studies did not involve endangered or protected species.

### Site characterization and sampling

The barks from three trees of *Anadenanthera peregrina* (L.) Speg. and of *Anadenanthera colubrina* (Vell.) Brenan were collected. The trees were growing in a remaining seasonal semi-deciduous mountain forest belonging to the *Mata Atlântica* bioma, located in the campus fields of the Federal University of Lavras, in the southern part of Minas Gerais, Brazil (21°14’S; 45°00’W, mean altitude 900 m). The climate is mesothermic with mild summers and dry winters (Cwb following the Köppen classification) with a mean annual temperature of 19.4°C and 1530 mm annual precipitation.

The trees were randomly selected and characterized: *A*. *peregrina* trees were 12–14 years old, with 12.5–14 cm diameter at breast height (dbh) and 5–7 m height; *A*. *colubrina* trees were 13–15 years old, with 11–14.5 cm dbh, and 6.5–8 m height.

The trees were harvested and the bark removed by separation from the wood by making an incision cut and pulling out. The samples used for analysis were taken from the lower part of the stem until 1.3 m of height.

### Anatomical characterization

The samples were impregnated with DP 1500 polyethylene glycol and transversal, tangential and radial microscopic sections were prepared from cambium to the outside [34, 35]. The proportion of cell types was determined using a 48-point grid on three images per sample on transverse sections from cambium to the outside. Sieve tubes were only quantified in the non-collapsed phloem. The length, width and wall thickness of fibres and sieve tube elements were measured in macerated samples.

The microscopic observations were made with a Leica DMLA optical microscope, the photomicrographs were taken with a Nikon FXA camera and the measurements done by using the Leica Qwin V 3.5.0 software. Terminology followed [36].

The macroscopic observation was made on the cross-section of the samples after surface sanding using a magnifying glass Leica MZ6 and photographed with a digital Samsung 10 MP L201 camera.

### Chemical composition

The bark samples were ground and the fraction 40–60 mesh used for chemical analysis.

A bark composite sample of the three trees per species was made and carefully homogenized before analysis. Determination of ash followed TAPPI standards (T 211 om-93), extractives were determined gravimetrically after successive Soxhlet extractions with dichloromethane, ethanol and water during 6 h, 16 h and 16 h, respectively (TAPPI Standard T204 om-88 and T207 om-93). The depolymerization and removal of suberin was made on the extractive-free samples using sodium methoxide in methanol [37].

The solid residue remaining after suberin depolymerization was used for determination of lignin and carbohydrates by acid hydrolysis. Acid insoluble (Klason) and acid soluble lignin were determined according to TAPPI T 222 om88 and TAPPI UM 250 standards, respectively. The composition of polysaccharides was evaluated after hydrolysis by determining the content in neutral monossacharides (rhamnose, arabinose, xylose, galactose, mannose and glucose) in the hydrolysate from the lignin analysis using high pressure ion-exchange chromatography with a pulsed amperometric detector (HPIC-PAD). The compounds were separated in a Dionex ICS-3000 system, with an Aminotrap plus Carbopac PA10 column (250 x 4 mm) using a linear gradient of NaOH and CH_3_COONa solution (0–20 min 18 mM NaOH; 20–25 min 50 mM NaOH+170 mM CH3COONa; 25–40 min 50 mM NaOH+170 mM CH3COONa) as eluent at a flow rate of 1mL/min; the column temperature was maintained at 30°C. All the chemical determinations were made on duplicate samples.

### Phenolic content of bark extracts

Extraction was carried out with ethanol-water (50%) for 60 min at 50°C using an ultrasonic bath and the extraction yield was calculated as the percent mass loss of the starting material.

The total amount of soluble phenolics was estimated by the Folin–Ciocalteu method using gallic acid as standard [38]. Total flavonoids were quantified by an aluminium chloride colorimetric assay using catechin as a standard [39] and the proanthocyanidins content (condensed tannins) was determined by the vanillin-H_2_SO_4_ method using catechin as a standard [40]. The experimental procedure is described in detail in [30].

### Antioxidant activity of bark extracts

The antioxidant activity was assessed by the DPPH free radical assay [41] following a procedure described before [30]. The antioxidant activity was expressed as IC_50_ values and also as in terms of Trolox equivalents (TEAC).

## Results

### Bark anatomy

**Fig 1:**
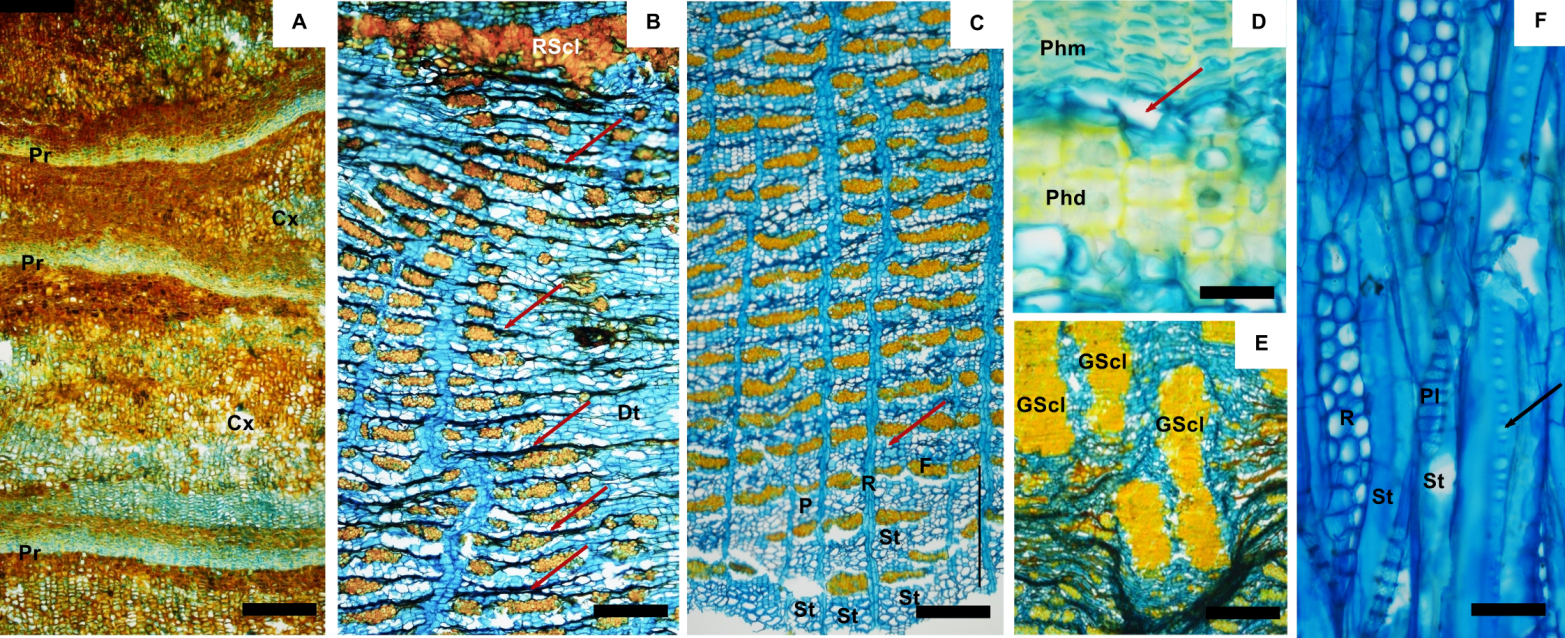
Bark of *A*. *peregrina*. A) rhytidome included sequentially periderms (Pr) interspersed with cortical cells (Cx); B) outer phloem showing the ring of sclerified cells (RSCL) which bounded inferiorly the cortex; dilatation tissue (Dt) from the wedged-shaped ray; ray (R) slightly distorted and dilated and stretched sieve tubes elements (arrows); C) Conducting phloem (black annotation) with tangential bands of the axial parenchyma cells (P), tangential bands of fibres (F) and sieve tube elements (St); nonconducting phloem marked by the collapsed of sieve tubes elements (arrow); D) Periderm with thick phellem cells (Phm), phellogen (arrow) and sclereids of the phelloderm cells (Phd); E) groups of sclereids (GScl) in dilatation tissue; F) Sieve tube elements (St) with compound sieve plates (PL) and lateral sieve areas (arrow); multiseriate rays (R) in tangential section. Scale bar: A, B, C, E = 150 μm; D, F = 25 μm.

**Fig 2:**
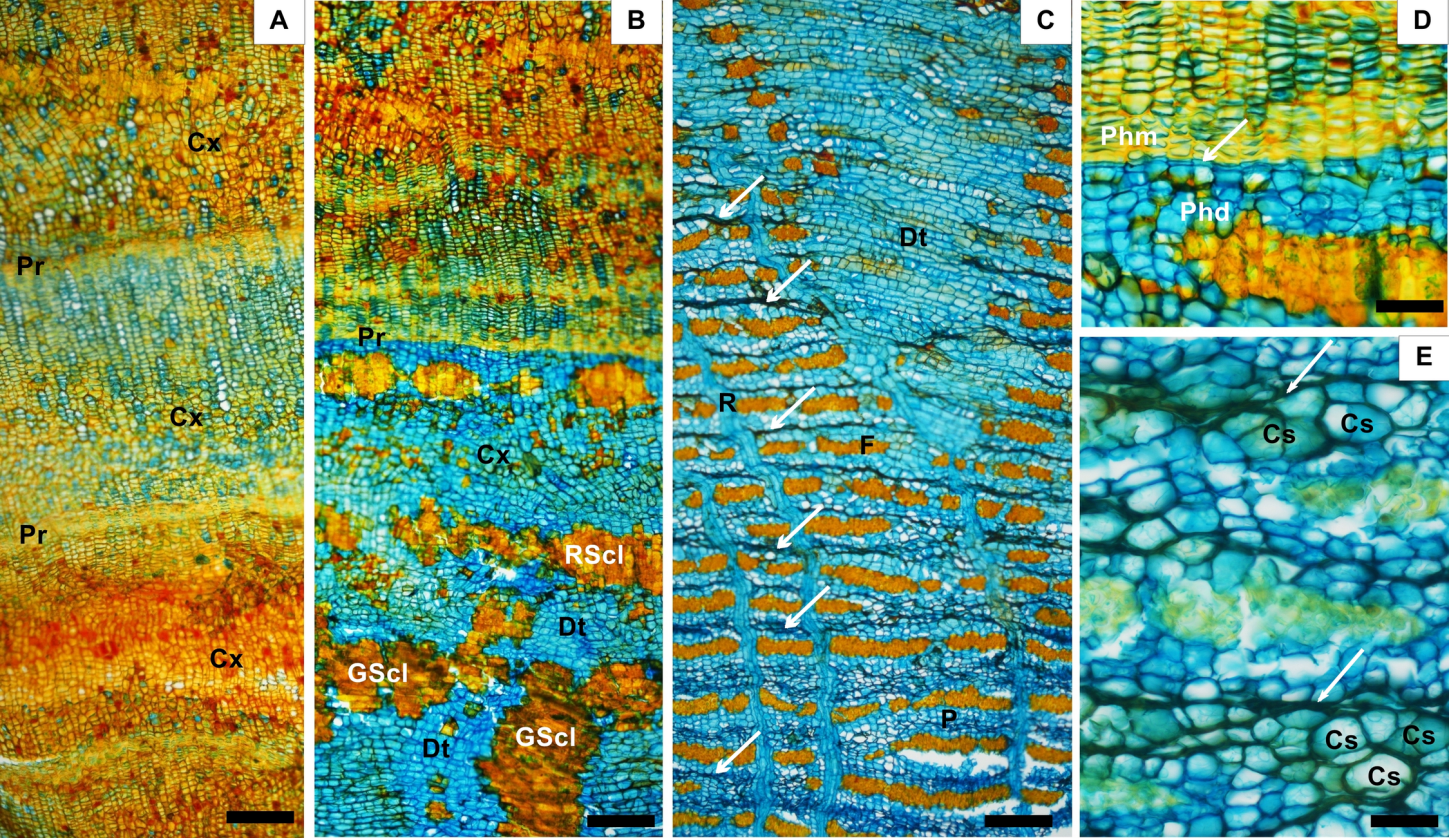
Bark of *A*. *colubrina*. A) rhytidome included sequentially periderms (Pr) interspersed with cortical cells (Cx); B) outer phloem near the periderm (Pr) showing the ring of sclerified cells (RSCL) which bounded inferiorly the cortex (Cx); dilatation tissue (Dt) and groups of sclereids (GScl); C) dilatation tissue (Dt) in form of wedged-shaped ray; bands of the axial parenchyma cells (P), tangential bands of fibres (F), rays (R) and stretched sieve tube elements (arrow) in the nonconducting phloem; D) Periderm with thick phellem cells (Phm), phellogen (arrow) and phelloderm cells (Phd) with thin walls and others uniformly thicken; E) Secretory cells (Cs) bounded by stretched sieve tube elements (arrow). Scale bar: A, B, C = 150 μm; D, E = 25 μm.

**Fig 3:**
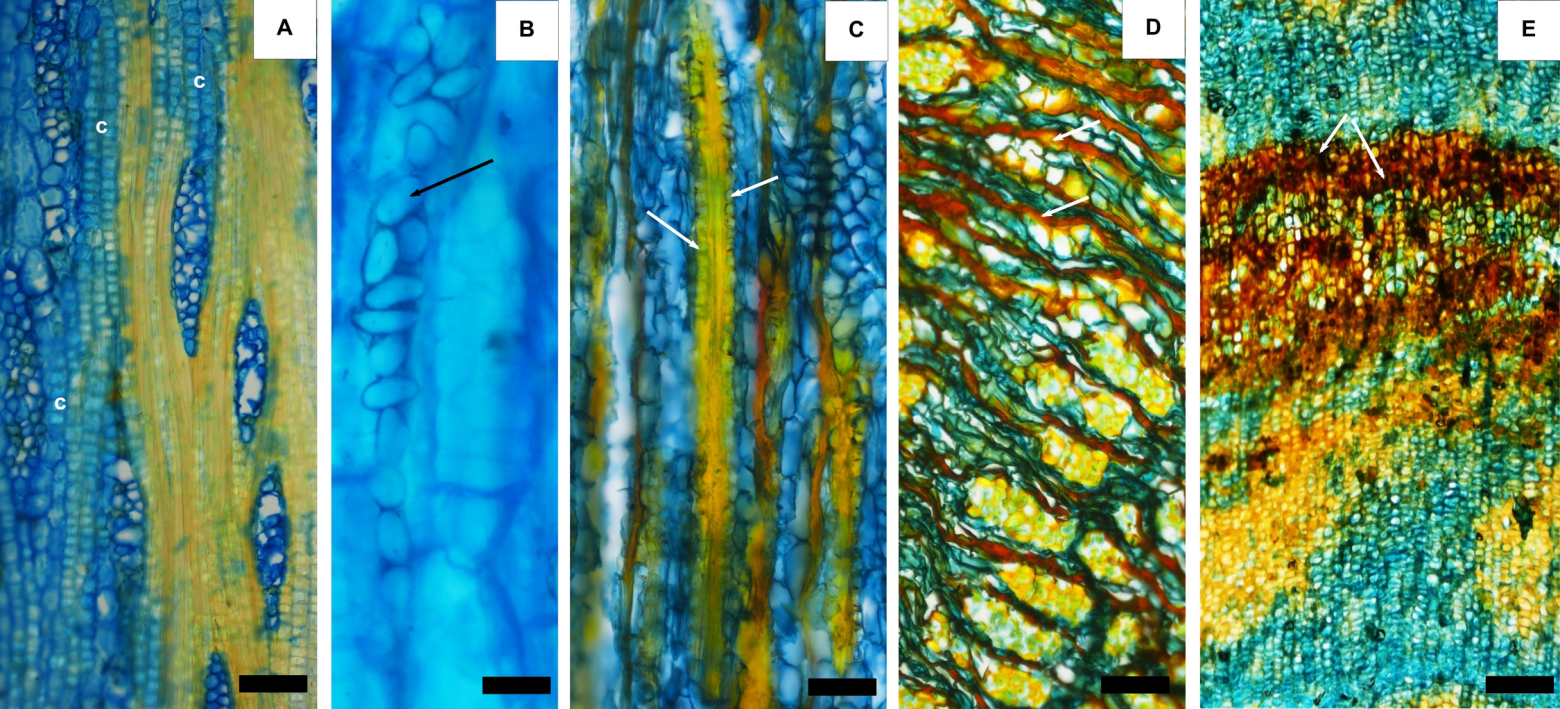
Mineral and organic inclusions in the bark. A) crystals (c) in chambered axial parenchyma cells; B) starch grains (arrow); C) crystals (arrows) adjacent to the fibres (F); Phenolic compounds in nonconducting phloem (arrows, D) and in rhytidome (arrows, E). Scale bar: A, C E = 50 μm; B = 25 μm; D = 150 μm.

The bark anatomy of *A*. *peregrina* and *A*. *colubrina* was similar. The anatomical bark features of both species are represented in Figs 1, 2 and 3. The macroscopic outer aspect of *A*. *peregrina* and A. *colubrina* barks was similar. The barks are brown and thick (6.5–17 mm and 5–12.1 mm respectively), wrinkled and irregularly scaly with sharp hardened outgrowths. The bark of *A*. *colubrina* was more difficult to remove from the trunk than that of *A*. *peregrina* that could be easily pulled out.

The bark included the rhytidome and the phloem; the nonconducting phloem was perfectly distinct from the conducting phloem by a lighter colour. Annual growth increments were not detected. In both species, the rhytidome was composed by several periderms that developed sequentially straight to undulated and were interspersed with cortical parenchyma cells (Figs 1A and 2A). In each periderm the phellem consisted of tabular cells with thick tangential walls and the phelloderm included layers of rectangular to round cells, some with thin walls and others uniformly thickened and even sclerified into sclereids (Figs 1D and 2D). These sclereids may give a certain pattern to the phelloderm either forming irregular groups or a somewhat concentering ring, more evident in *A*. *peregrina*

The cortical parenchyma cells, derived from the cortex i.e. the phellogen developed in the cortex, were arranged without intercellular spaces, particularly obvious in the outgrowths of *A*.*colubrina* (Fig 2A), and could be confused as phellem cells of the periderm. In fact the periderm of *A*. *peregrina* and *A*. *colubrina* included only a small proportion of suberized phellem cells.

Below the innermost periderm, there was a zone with cortical-like cells, here called "cortex", where cells were dilated by tangential expansion and also sclerified forming groups of sclereids (Fig 2B); this area is inner bordered by a ring of sclerified cells that make the transition to the phloem (Figs 2B and 3B).

*A*. *peregrina* and *A*. *colubrina* bark structure was similar also concerning: 1) the demarcation between non-conducting and conducting phloem that was gradual and marked by collapsed sieve tube elements; 2) phloem stratification; 3) type and position of sieve plates; 4) dilatation tissue restrict to the rays; 5) arrangement and proportion of sclereids; and 6) presence of secretory cells.

The conducting phloem was narrow (about 20 cell rows) and included the sieve-tube elements with turgid companion cells, axial and radial parenchyma cells and fibers (Fig 1C). The sieve-tube elements (corresponding to approximately 2% in *A*. *peregrina* and *A*. *colubrina*) were solitary or in small groups of 2 or 3, and scattered among axial parenchyma cells. The sieve plates are compound scalariform and inclined, with 5–10 sieve areas (Fig 1F); lateral sieve tube walls were observed with pores clustered in sieve areas (Fig 1F). The diameter and length of sieve-tube elements were similar in both species: 25.0–28.5 μm and 275–326 μm in *A*. *peregrina* and 27.3–31.9 μm and 320–324 μm *in A*. *colubrina*, respectively; Costa et al. (1997) found longer conducting cells in adult trees of *A peregrina* varying from although with similar diameters (367–502 μm and 18–30 μm respectively).

The axial parenchyma cells (34% in *A*. *peregrina* and 23% in *A*. *colubrina*) were round in more or less continuous bands of 2–3 cells in *A*. *peregrina* and 2–6 cells in *A*. *colubrina* that alternate with continuous tangential bands of fibers (Figs 1C and 2C).

The fibers (35% in *A*. *peregrina* and 24% in *A*. *colubrina)* were rounded polygonal in transverse section and arranged in tangential bands (3–4 cells wide in *A*. *peregrina* and 2–3 cells wide in *A*. *colubrina*), interrupted by the rays. The fibers were slightly longer and thinner in *A*. *peregrina* (1201 μm long and 6.4 μm thick) than in *A*. *colubrina* (1187 μm and 6.8 μm).

The rays (13% in *A*. *peregrina* and 14% in *A*. *colubrina*) were non-storied; mostly multiseriate (2–4 cells), homocelular with procumbent ceIls (Fig 1F), and more or less straight until the beginning of the dilatation growth. The number of cells in the rays was the same in both species (7–28 in height and 6–12 cells/mm).

The non-conducting phloem is characterized by the collapsed sieve-tube elements (Figs 1B, 1C and 2C) and the dilatation growth. In *A*. *peregrina* and *A*. *colubrina* the dilatation is evident mainly in the outer phloem and results mostly through the anticlinal ray cell division, giving rise to a slightly to dilated rays (Figs 1B, 2B and 2C); in minor extent axial and radial parenchyma cells also undergo some tangential dilatation.

The dilatation tissue represented about 12% in *A*. *peregrina* and 29%.in *A*. *colubrina*. Clusters of sclereids occured resulting in conspicuous nodules of irregular or radial alignment (Figs 1E and 2B) with a characteristic pattern. Sclereids (4% in *A*. *peregrina* and 8% in *A*. *colubrine)* appeared isodiametric with a polylamellated and pitted wall. Sclereids constitute the mechanical support for bark radial growth in addition that given by the fibers (higher in *A*. *peregrina*).

In *A*. *peregrina* and *A*. *colubrina*, prismatic crystals occurred in chambered axial parenchyma cells (Fig 3A) and also adjacent to fibrous bands (Fig 3C). Single crystals were frequent in sclerified cells of the phelloderm, mostly in *A*. *colubrina*.

Starch grains were observed in parenchyma cells of *A*. *colubrina* (Fig 3B) Phenolic deposits of brown colour were particularly abundant through the bark, mainly in the rhytidome i.e. phellem, phelloderm and cortex, and in the non-conducting phloem, arranged in tangential rows or groups (Fig 3D and 3E). In both species, enlarged parenchyma cells (secretory cells) were observed in nonconducting tissue arranged in groups (Figs 1F and 2E).

### Chemical composition

Table 1 summarizes the chemical composition and the polysaccharides composition of the barks of *A*. *peregrina* and *A*. *colubrina*. The chemical composition of the bark of both species was similar. The content in extractives was very high at 29%, corresponding mainly to polar extractives soluble in ethanol and water that represent 85% and 90%, respectively of the total extractives. The suberin content was low corresponding on average to 2.5% of the barks. Lignin content was on average 18.9% and polysaccharides 40%. The monosaccharide composition showed a predominance of glucose corresponding on average to 82% of the neutral monosaccharides.

**Table 1:** Summative chemical composition (% of dry bark), monosaccharide composition (% of total neutral monosaccharides) of the barks of *Anadenanthera peregrina* and *Anadenanthera colubrina*.

	*A*. *peregrina*	*A*. *colubrina*
Chemical composition (% of bark)		
Ash	8.2	7.7
Extractives		
Total	28.8	29.3
Dichloromethane	3.0	2.8
Ethanol	21.0	22.5
Water	4.7	4.1
Suberin	2.4	2.6
Lignin		
Total	18.8	18.9
Klason lignina	16.4	16.8
Soluble lignin	2.8	2.1
Polysacharides	41.8	41.5
Monosaccharide composition (% of total neutral monosaccharides)		
Rhamnose	0.1	0.3
Arabinose	2.1	3.2
Xylose	8.7	8.9
Mannose	2.1	2.3
Galactose	4.1	4.1
Glucose	82.9	81.2

The hemicelluloses included mostly xylan i.e. xylose and arabinose accounted on average for 8.8% and 2.6% of the total neutral monomeric sugars, respectively, but also galactomannan i.e. galactose and mannose represented on average 4.1% and 2.2%, respectively. The glucose/xylose ratio was high at 9.5 for *A*. *peregrina* and 9.1 for *A*. *colubrina*. Both barks had a large mineral content: 8.2% for *A*. *peregrina* and 7.7% for *A*. *colubrina*.

### Phenolic content of bark extracts

The yield of ethanol-water extraction and the phenolic composition and anti-oxidant activity of the bark extracts are given in Table 2. The extraction yield of 22.8% and 25.4% for the barks of *A*. *peregrina* and *A*. *colubrina* respectively was very similar to the content of the polar extractives determined by sequential solvent extraction (Table 1).

**Table 2:** Ethanol-water extraction yield (% of dry bark), total phenolic content, tannins and flavonoids content and antioxidant activity of the barks of *Anadenanthera peregrina* and *Anadenanthera colubrina*.

	*A*. *peregrina*	*A*. *colubrina*
Extraction yield (% of bark))	22.8	25.4
Total phenolic content (mg GAE g^-1^ of extract)	583.0	682.0
Flavonoids (mg catechin g^-1^ of extract)	148.4	445.3
Tannins (mg catechin g^-1^ of extract)	586.9	97.5
Antioxidant capacity TEAC (mg Trolox g^-1^ of extract)	237.6	268.6
Antioxidant capacity TEAC (mg Trolox g^-1^ of bark)	54.3	63.7
IC_50_ values (μg extract ml^-1^)*	13.1	13.5

There was a substantial difference in the extract composition between the two species. The total phenolic content was high, especially for *A*. *colubrina* (682 mg GAE/g extract vs. 583 mg GAE/g extract) that contained also substantially more flavonoids (445 mg CE/g extract vs. 148 mg CE/g extract). On the contrary, the tannin content was much higher in the *A*. *peregrina* bark extract (587 mg CE/g extract vs. 98 mg CE/g extract). The antioxidant activity of both bark extracts was similar corresponding on average to 253 mg Trolox g^-1^ extract or 59 mg Trolox g^-1^ of bark.

## Discussion

### Anatomical features

*A*. *peregrina* and *A*. *colubrina* could be differentiated by the adhesion of the bark to the cambium and xylem since the bark of *A*. *peregrina* could be pulled out easily. This difference, which should be related with cambial features and has been referred for other species e.g. for Eucalyptus spp. [28].

The bark anatomy of both *Anadenanthera* species was very similar. [15] also observed little anatomical variation in the bark of different genus of Mimosaceae subfamily. The bark of *A*. *peregrina* and *A*. *colubrina* showed overall common features: a conspicuous scaly rhytidome with several periderms with little phellem development interspersed by cortical tissues. This type of rhytidome with successive periderms including bands of cortical parenchyma was described by [16] for *A*. *peregrina* and other species of the same family i.e. *Bowdichia virgilioides*. These cortical parenchyma cells were particularly obvious in the outgrowths of *A*.*colubrina* (Fig 2A) and their form and radial alignment could mistake them with phellem cells of the periderm. [15] also referred in cork of Mimosaceae and above the scale, the presence of small cells which preserve their embryonic shape and meristematic state and may be able to divide periclinally resembling phellogen cells; the little suberization of the cork was also noticed by the same author.

The cortex below the innermost periderm featuring a more or less complete ring of irregularly shaped sclereids was also noticed by [14], [15] and [16]. The phloem included a narrow conducting region with anatomical features that were similar in both species and that in general agree with the observations of [16] for the Mimosoideae. The nonconducting phloem showed a conspicuous dilatation tissue formed by parenchyma cell division and expansion, resulting from the phloem adjustment to tree radial growth [36] and are in agreement with observations for the Leguminoseae by (15) and [16].

Thick walled sclereids were present in large proportion, especially in *A*. *colubrina* often including large prismatic crystals. The presence of crystals accompanies sclerefication and contributes to the mechanical strength of the bark [42, 15].

Numerous crystals were observed in the phloem of both species namely in the axial parenchyma close to the fibres (Fig 3A and 3C). Crystals in the axial parenchyma seem to be a characteristic of the Mimosaceae subfamily [15] and were described for *A*. *peregrina* [43, 16]. Although some authors refer that crystals may have a diagnostic value to differentiate species within a genus [44, 45, 46], [47] considers that variability is also large between individuals in the same species. Starch grains were observed in parenchyma cells of *A*. *colubrina* (Fig 3B) and their presence in the axial parenchyma in the majority of the studied species examined by [16].

A conspicuous feature of these barks was the presence of extensive phenolic deposits in e.g. tanniferous cells especially in the rhytidome and in the non-conducting phloem (Fig 3D and 3E) that seemed overall more abundant in *A*. *peregrina* bark. This is clearly in agreement with the traditional use of these barks as a tannin source, and with the chemical features as discussed further on.

Overall the anatomical structure of the barks of *A*. *peregrina* and *A*. *colubrina* was very similar and the small differences that were observed do not allow a bark based taxonomic differentiation of both species.

### Chemical composition

There are no previous studies and references on the chemical composition of the barks from *A*. *peregrina and A*. *colubrina*. It is however known that they contain a large amount of tannins, especially the bark of *A*. *peregrina*, and that they are used in the leather and dyeing industries [17, 18, 19].

The determination of extractives (Table 1) confirms the high content of polar extractives that can be extracted by an ethanolic aqueous solution (Table 2). The microscopic observations also showed the high amount of deposits and inclusions that are a conspicuous feature of the bark structure of these species (Fig 3D and 3E). It was also confirmed that the bark extract of *A*. *peregrina* contains a much higher tannin content that represents about 59% of the extract (in catechin equivalents) while tannins in the bark of *A*. *colubrina* represent only about 10% of the extract (Table 2).

The valorization of *A*. *peregrina* bark is therefore certainly possible for production of tannins that will represent approximately 17% of the bark (173.3 mg CE/g bark). The bark extract of *A*. *colubrina* is very rich in total phenolics (ca. 68% in GAE of the extract) that include mainly flavonoids (45% in CE of the extract) (Table 2). Flavonoids are also valued as bioactive supplements and therefore their extraction from *A*. *colubrina* bark represents also a potential valorization route. However the antioxidant properties of the bark extracts of both species are only of moderate intensity with an average IC_50_ value of 13 mg ml^-1^ compared with 2 mg ml^-1^ for Trolox (Table 2).

Barks from other species may also contain a high proportion of extractives. For instance 28.1% and 28.3% for *Eucalyptus grandis x urophyla* and *E*. *grandis*, respectively [48] or 26.7% for *Pseudotsuga menziesii* [30]. The phenolic content of bark extracts is very variable between species but usually below the values found here. For instance 386, 347 and 204 mg GAE/g extract in barks of *E*. *grandis*, *E*. *urograndis* and *E*. *maidenii*, respectively [49], 80, 78, 66, 66, 38 and 25 mg GAE/g extract in barks of *Acacia nilotica*, *Acacia catechu*, *Albizia lebbeck*, *Senna tora*, *Saraca asoca* and *Caesalpinia sappan*, respectively where the flavonoid content ranged 4–22 mg CE/g extract [50], 211–551 mg GAE/g extract in barks of various *E*. *urophylla* hybrids [30] or 93, 165, 120 and 120 mg GAE/g extract in barks of *Eugenia jambolana*, *Acacia nilotica*, *Azadirachta indica* and *Terminalia arjuna*, respectively where the flavonoid content ranged 21–49 mg CE/g extract [51]. Flavonoids represent 75 mg quercetin equivalent/g extracts in the bark of *Delonix elata* [52]. Tannins in the barks of *Alnus incana* and *A*. *glutinosa* represent respectively 43% and 34% of the extract [53].

Since the barks of *A*. *peregrina* and *A*. *colubrina* have a small proportion of phellem in their periderms, their content in suberin is low (Table 1). Suberin is a typical structural component of bark periderms where its presence is specific to the wall of phellem cells for which it is a chemical fingerprint [31, 54, 55]. Therefore when the proportion of phellem in a bark is small, then the content in suberin is also small. This has been shown for several species e.g. *Pinus sylvestris* and *Picea abies* [56], *E*. *globulus* [57] or *Tectona grandis* [33].

The main structural chemical components of the barks of *A*, *peregrina* and *A*. *colubrina* are lignin, which represented 31.0% and 31.1% respectively of the extractive and ash-free barks, and by cellulose and hemicelluloses. Xylan and galactoglucomannans should be the major hemicelluloses, while glucose was the predominant monosaccharide obtained after acid hydrolysis (Table 2). It should be noted that only neutral monosaccharides were determined and it is to expect the presence in the hemicelluloses of acetyl groups and uronic acids.

The high mineral content of both barks should result from the frequent presence of crystals, presumably of calcium oxalate, that are clearly observed namely in the cells of the axial parenchyma (Fig 3A and 3C).

## Conclusions

The barks of the two species of the genus *Anadenanthera* (*A*. *peregrina* and *A*. *colubrina*) are very similar in relation to the structural arrangement, the type and morphology of cells, and tissue distribution in phloem, rhytidome and periderms. Both species were distinguished by the sclereids in the phelloderm forming a more obvious ring in *A peregrina* and by the type of cortical parenchyma cells in the rhytidome in *A*. *colubrina* that resemble phellem cells. However the bark anatomy cannot per se be used as a taxonomic indicator.

The chemical composition of *A*. *peregrina* and *A*. *colubrina* barks was studied for the first time. The most striking characteristic is the very high content of polar extractives rich in phenolic compounds and with good antioxidant activities. Significant differences were found in the content of polar extractives with *A*. *peregrina* bark showing a higher content of tannins and *A*. *colubrina* a higher content of total phenolics and flavonoids.

Their extraction will represent an important valorization of these barks and therefore contribute to improve the overall economic potential and sustainability of *A*. *peregrina* and *A*. *colubrina*.
